# Population genetics of the European rabbit along a rural-to-urban gradient

**DOI:** 10.1038/s41598-020-57962-3

**Published:** 2020-02-12

**Authors:** Madlen Ziege, Panagiotis Theodorou, Hannah Jüngling, Stefan Merker, Martin Plath, Bruno Streit, Hannes Lerp

**Affiliations:** 1 University of Potsdam, Plant Ecology and Nature Conservation, Am Mühlenberg 3, D-14476 Potsdam, Golm Germany; 2 Martin-Luther-University Halle-Wittenberg, Institute of Biology, General Zoology, Hoher Weg 8, D-06120 Halle, Saale Germany; 3 Senckenberg Gesellschaft für Naturforschung, Clamecystraße 12, D-63571 Gelnhausen, Germany; 4 State Museum of Natural History Stuttgart, Department of Zoology, Rosenstein 1, D-70191 Stuttgart, Germany; 5 Northwest A&F University, College of Animal Science and Technology, Yangling, Shaanxi 712100 China; 6 University of Frankfurt, Department of Ecology & Evolution, Max-von-Laue-Str. 13, D-60438 Frankfurt am Main, Germany; 7 Museum Wiesbaden, Natural History Collections, Friedrich-Ebert-Allee 2, D-65185 Wiesbaden, Germany

**Keywords:** Genetics, Conservation biology

## Abstract

The European rabbit (*Oryctolagus cuniculus*) is declining in large parts of Europe but populations in some German cities remained so far unaffected by this decline. The question arises of how urbanization affects patterns of population genetic variation and differentiation in German rabbit populations, as urban habitat fragmentation may result in altered meta-population dynamics. To address this question, we used microsatellite markers to genotype rabbit populations occurring along a rural-to-urban gradient in and around the city of Frankfurt, Germany. We found no effect of urbanization on allelic richness. However, the observed heterozygosity was significantly higher in urban than rural populations and also the inbreeding coefficients were lower, most likely reflecting the small population sizes and possibly on-going loss of genetic diversity in structurally impoverished rural areas. Global *F*_ST_ and *G*′_ST_-values suggest moderate but significant differentiation between populations. Multiple matrix regression with randomization ascribed this differentiation to isolation-by-environment rather than isolation-by-distance. Analyses of migration rates revealed asymmetrical gene flow, which was higher from rural into urban populations than vice versa and may again reflect intensified agricultural land-use practices in rural areas. We discuss that populations inhabiting urban areas will likely play an important role in the future distribution of European rabbits.

## Introduction

As cities expand worldwide, urban areas will double in size, cover approximately 10% of Earth’s landmass, and host around 5 billion people by 2030^1^. Urbanization has been identified as one of the major global threats to biodiversity in the Anthropocene^2,3^ and a major driver of evolutionary change^4^.

Urbanization leads to the destruction of natural habitats and creates new, highly fragmented landscapes with increased impervious surfaces, as well as air, water, light and noise pollution^5,6^. Only organisms that are able to cope with (or adapt to) the novel set of abiotic and biotic conditions colonize urban ecosystems^4,7^. Understanding how the expansion of cities—as a major component of global environmental change—affects the distribution of biodiversity as well as evolutionary change within species has become increasingly important for the conservation of biodiversity on Earth^4,8^. At the same time, cities can be viewed as unintended ‘large-scale experiments’^8^, providing scientists with the unique opportunity to elucidate general principles of ecological dynamics and study evolutionary processes.

Using molecular markers and spatial ecological tools, a number of studies addressed the question of how evolutionary processes—including novel mutations, genetic drift, gene flow, and selection—operate in cities^4,9^. Two widely studied evolutionary processes in urban wildlife populations are genetic drift and gene flow^4^. The strength of genetic drift is predicted to increase with increasing urbanization^4^. Most of the wildlife in cities occurs within the few remnant semi-natural patches and/or other human-made green spaces such as parks, botanical and private gardens^3,4^. These areas are likely to harbour small and isolated populations, surrounded by strong barriers to dispersal (e.g., streets, highways and buildings). Empirical evidence for reduced dispersal and gene flow among urban populations comes from studies on various animal and plant species^4^. This is true even over short geographic distances and for otherwise very mobile species such as song sparrows (*Melospiza melodia*)^10^, wren-tits (*Chamaea fasciata*)^11^ and red foxes (*Vulpes vulpes*)^12^. Isolation of populations, coupled with small population sizes, will exacerbate the effects of genetic drift and can result in reduced genetic diversity within and greater genetic differentiation among urban populations, as has been shown for a number of species, including the white-footed mouse (*Peromyscus leucopus*)^13^, the yellow toadflax (*Linaria vulgaris*)^14^ and the fire salamander (*Salamandra salamandra*)^15^.

Nonetheless, some animal and plant species are thriving in cities and do not seem to follow the aforementioned patterns of altered meta-population dynamics and reduced genetic variability^16–18^. For instance, increased urbanization does not reduce gene flow nor genetic diversity in the downy yellow viole (*Viola pubescens*)^19^, orchid bees (*Euglossa viridissima* and *E. dilemma*)^20^, the spotted salamander (*Ambystoma maculatum*)^21^, the west coast ctenotus (*Ctenotus fallens*)^22^, the ornate box turtle (*Terrapene ornata*)^23^, the red-tailed bumblebee (*Bombus lapidarius*)^24^ and the Eurasian tree sparrow (*Passer montanus*)^25^. This is not surprising, as the effects of urbanization are likely dependent on the form and intensity of land use, the spatial scale of investigation, and especially ecological attributes of the studied taxonomic group (e.g. dispersal ability, reproductive biology, diet and historical demography). Urban habitats can sometimes harbour large populations of certain species and do not always hinder, but might rather facilitate gene flow. Green spaces within the urban ecosystem may promote gene flow^26–28^ and may act as sources of genetic diversity^29^. Furthermore, while rivers and roads in cities represent barriers to some species^30,31^ they may function as corridors for dispersal and gene flow in others^32,33^.

Several factors—including landscape processes—can lead to a reduction of gene flow and thus, genetic differentiation between populations^34^. Under isolation-by-distance (IBD), geographic (but not environmental) distances predict genetic differentiation, as the likelihood of dispersal decreases when the distance between populations increases^35,36^. Moreover, the extent of gene flow can be a correlate of environmental (not geographic) distances between populations, resulting in patterns of isolation-by-environment (IBE)^37,38^. IBE may result from various mechanisms, including selection and local adaptation, as well as non-random dispersal and gene flow, and could be as common as IBD across environmental gradients^35^. In a study of white-footed mouse populations in the New York City metropolitan area, Munshi-South *et al*.^13^ used IBD and IBE modelling to demonstrate that urbanization (i.e., IBE) drives genetic differentiation to a greater extent than geographic distances (i.e., IBD).

Here, we examine whether and how urbanization influences genetic diversity, dispersal/gene flow, and genetic differentiation in populations of the European rabbit (*Oryctolagus cuniculus*) occurring in and around the city of Frankfurt am Main in Germany. European rabbits are native to the Iberian Peninsula^39^, from where they reached Western Europe since Roman times^40^. While European rabbits are considered a multifunctional keystone species in their native range, crucial in maintaining the organization and diversity of local ecological communities^41^, they are also one of the most successful invasive mammals world-wide, often with dramatic negative effects on local biodiversity, ecosystems, and the economy^42–44^. Currently, populations within the species’ native range (and throughout Europe) are dramatically declining^45^, most likely due to habitat loss as a result of intensified agricultural practices in combination with diseases like myxomatosis and rabbit calicivirus, as well as human-induced mortality^45^. Rabbit populations in several German cities appeared to be largely unaffected by this decline and were reported to reach high densities^46–48^. Urban habitat diversity and the increased availability of suitable sites for burrow construction in parks and gardens are among the hypothesized drivers underlying this pattern^49^. However, a vast decrease in hunting bag records within German cities such as Frankfurt indicate that also urban rabbit populations are affected by the overall population decline (personal communication with local hunters). The European rabbit is considered as an ‘urban adaptor’—a species that may utilize human resources and survive in human-dominated areas, but does not necessarily receive an added benefit from living with humans^17^.

Based on previous studies^46,49^, we predicted similar or even increased levels of genetic diversity along the rural-to-urban gradient (i.e., from rural sites towards sites situated in the city centre). Second, we predicted that both geographic distances (IBD) and environmental distances (IBE) predict the amount of gene flow, which should decrease with increasing geographic and environmental distances.

## Methods

### Study populations and sampling sites

According to city archive records, rabbit populations have been established in Frankfurt am Main at least since the 1930s (Stadtarchiv Frankfurt). In Römer-Büchner (1827) we found indications that rabbits were present close to the city of Frankfurt am Main already in the 19^th^ century^50^. Frankfurt is the largest city in the state of Hesse and the fifth-largest city in Germany^51^. About 736,000 people live in the city’s administrative boundaries and about 2.4 million in its functional urban area, with an average density of 3,000 inhabitants per square kilometer^51^. Nonetheless, Frankfurt is considered a ‘green city’ since more than 50% of the area within the city limits are protected green areas (e.g., forest, parks and gardens^51^).

**Figure 1:**
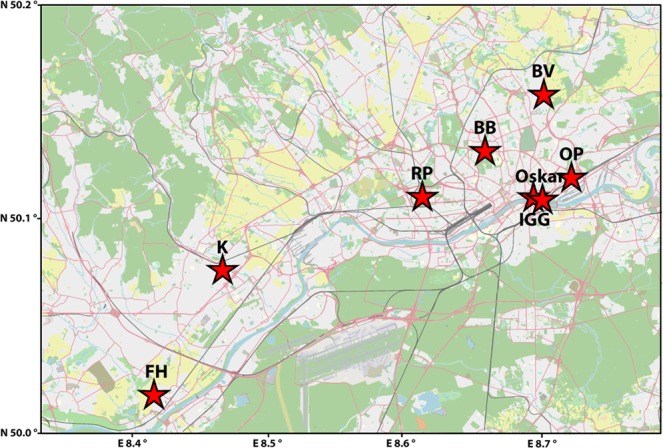
Map showing the location of our eight study sites in and around Frankfurt am Main. *BV*, Bad Vilbel; *FH*, Flörsheim; *K*, Kriftel; *OP*, Ostpark; *RP*, Rebstockpark; *IGG*, innerer Grüngürtel; *Oskar*, Oskar-von-Miller Straße; *BB*, Bundesbank. Map modified, © OpenStreetMap contributors, CC BY-SA 2.0. https://creativecommons.org/licenses/by-sa/2.0/.

We chose our eight study sites along a rural-to-urban gradient and included two parks in the city centre (former rampart areas; IGG, Oskar) that are surrounded by a high density of roads and human infrastructure, three parks located at the former periphery of the administrative district in Frankfurt with a medium density of roads and human infrastructure (OP, RP, BB), and three adjacent rural areas with a low density of roads and human infrastructure (BV, FH, K; Fig. 1; Table 1). Distances between our sampling sites ranged between 1 km and 22.3 km (Fig. 1; Supplementary Table S1). We sampled a total of 139 rabbits from the eight sites between October 2012 and March 2013 in cooperation with local hunters and as part of the regular hunting scheme conducted and approved by authorized city hunters in accordance with the Hessian hunting laws (hunting licence 1000250221; Fig. 1; Table 1). All samples were tissue material with the exception of three, which were hair samples.

**Table 1:** Average allelic richness (*A*_R_; based on rarefaction), observed (*H*_O_) and expected heterozygosity (*H*_E_), inbreeding coefficient (*F*_IS_), results of Wilcoxon sign rank tests (*p*-values for one-tailed tests of excess heterozygosity) testing for bottlenecks under the two-phase (TPM) and stepwise mutation model (SMM), as well as the ‘urbanity index’ for our eight study populations of European rabbits in and around Frankfurt am Main, excluding loci *sat12* and *7L1F1*.

Population	Geolocation coordinates		*N*	*A*_R_	*H*_O_	*H*_E_	*F*_IS_	TPM	SMM	Urbanity index
Bad Vilbel (*BV*)	N 50°9.418	E 8°41.820	11	2.640	0.470	0.520	0.095	0.421	0.421	−1.329
Flörsheim (*FH*)	N 50°0.998	E 8°24.838	10	2.860	0.520	0.600	0.132	0.125	0.156	N.A.
Kriftel (*K*)	N 50°4.585	E 8°28.010	14	2.870	0.500	0.590	0.154	0.125	0.156	−1.194
Ostpark (*OP*)	N 50°7.251	E 8°43.364	25	2.750	0.600	0.570	−0.050	0.273	0.421	−0.464
Rebstockpark (*RP*)	N 50°6.674	E 8°36.773	29	2.510	0.540	0.500	−0.070	0.156	0.230	−0.692
Innerer Grüngürtel (*IGG*)	N 50°6.673	E 8°41.608	17	2.780	0.610	0.580	−0.050	0.191	0.320	0.279
Oskar-von-Miller-Straße (*Oskar*)	N 50°6.515	E 8°41.880	7	2.550	0.580	0.500	−0.164	0.628	0.679	1.126
Bundesbank (*BB*)	N 50°7.970	E 8°39.524	25	2.840	0.610	0.580	−0.052	0.125	0.230	−0.467
Mean across populations			17.25	2.725	0.553	0.555	−0.0006			

Since the degree of urbanization does not necessarily decrease continuously towards the outskirts of a city^52^, we refrained from categorizing our sites into distinct classes and instead calculated a continuous variable, the “urbanity index”^46,49^. To this end, we incorporated several variables related to the degree of both, anthropogenic disturbance and landscape alterations. First, we quantified the number of residents located within a radius of 500 m from each site (*Einwohnermeldeamt* of the city of Frankfurt am Main). Additionally, we estimated the intensity of disturbance by humans (i.e., pedestrians and bikers) and leashed or unleashed dogs during the main activity period of the rabbits at dawn and dusk. Locations to quantify disturbance were randomly selected for each study site using the ArcMap Random Point Generator in ESRI ArcGIS 10 (ESRI, 2011). The appropriate number of transect belts within study sites was determined in relation to the size of the area. These random locations were used as starting points to draw a virtual transect line of 25 m towards the North. All pedestrians, bikers and dogs crossing this transect line were counted for three minutes every 30 min. In total, 20 counts per site were performed on five consecutive days. Furthermore, we quantified the proportion of artificial ground cover (e.g., streets, buildings) within a radius of 500 m from each of our study sites using a geographic information system (ArcGIS 10). We log‐transformed our data and subjected all variables to a principal component analysis in SPSS v. 13.0 for windows. The first principal component explained 80.87% of the variance and was used as a metric of the ‘degree of urbanity’^46,49^.

### DNA extraction and microsatellite amplification

We extracted high molecular weight DNA using the Qiagen DNeasy Blood & Tissue Kit® following the manufacturer’s instructions (Qiagen, Hilden, Germany). We stored DNA samples at −20 °C until PCR-amplification at ten previously described microsatellite loci (Supplementary Tables S2 and S3), using an MJ Research PTC-225 thermocycler (BioRad, California, countryUSA). Multiplex PCRs were performed in 12.5 µl reaction volumes consisting of 6.25 µl 2 × Qiagen multiplex PCR master mix and 1 µl primer mix (5 pmol/l for each primer according to the multiplex primer mix shown in Supplementary Table S4), 1 µl Q-solution, 2.25 µl DNase-free water and 2 µl template DNA (25–50 ng). We electrophoresed all PCR products on a Beckman Coulter CEQ. 2000 automated capillary sequencer (Beckman Coulter, California, USA). Samples were screened using Genome Lab GeTX 10.2 software (Beckman Coulter, Indianapolis, USA) and alleles scored manually. We independently genotyped all samples two times and, in the case of conflicting results, ran two additional amplifications. We inferred the correct genotype from the majority of the four replicates.

### Evaluating microsatellite markers, genetic diversity, bottlenecks and genetic structure

We inspected our microsatellite data for the presence of null-alleles, large allele drop-out, and mis-scoring due to ‘stutter-bands’ using MICROCHECKER v. 2.2.3^53^. Tests for Hardy–Weinberg equilibrium and linkage disequilibrium were performed in GENEPOP web v. 4.0.10^54^ using Bonferroni corrected *α*-levels^55^ to reduce Type I errors in multiple testing. To check for the potential occurrence of related individuals in the dataset, we used GenAlEx v. 6.5^56^, with which we calculated relatedness coefficients. The analysis did not detect any pairs of individuals being highly related and so all genotyped individuals were treated as independent samples. Using the R package *diversity* v. 1.9.90^57^ we calculated the number of alleles per locus, rarefied allelic richness (*A*_R_), observed (*H*_o_) and expected heterozygosities (*H*_E_), as well as the inbreeding coefficient (*F*_IS_) and pairwise *F*_ST_ and *G*′_ST_-values^58^ between all eight sampling sites using 1,000 bootstrap iterations.

To investigate whether a given population has undergone a recent bottleneck we used the BOTTLENECK v.1.2.02 software^59^. BOTTLENECK compares the observed heterozygosity at each locus with the expected heterozygosity assuming mutation-drift equilibrium using a Wilcoxon signed-rank test. An excess in heterozygosity suggests recent population contraction (bottleneck) and heterozygosity deficit suggests recent population expansion. We used a stepwise mutation model (SMM) and a two-phase model (TPM; 95% single-step mutations and 5% multiple-step mutations as recommended by the authors of the software) followed by 10,000 simulation iterations.

To infer the number of genetic clusters we used *K-means* clustering implemented in the *find.clusters* function within the R^60^ package *adegenet*^61^. This function first transforms the data using a principal component analysis and then it runs successive *K-means* clustering with increasing number of clusters (*K*). We performed the analysis without prior information on group membership of individuals and we assess the optimal number of groups using the Bayesian information criterion (BIC). To assess the level of admixture within sites, we used discriminant analysis of principal components (DAPC)^62^, implemented in the R package *adegenet*^61^. This approach transforms multilocus genotype data using principal component analysis to derive uncorrelated variables that serve as input for discriminant analysis. In the assessment of population structure, the discriminant analysis aims to maximize among-group variation and minimize within-group variation. In contrast to Bayesian clustering methods, DAPC does not require a population genetic model (Hardy–Weinberg or gametic equilibrium) and performs better at handling hierarchical structure or cline variation caused by isolation-by-distance^62,63^. The analysis was performed both with and without prior information on individual populations. When group priors were used, we used a barplot (*compoplot* command in *adegenet*) to visualize membership of individuals to different clusters.

### Migration rates

To estimate recent migration rates (*m*) between populations, we used BayesAss v. 1.3^64^, which uses a Bayesian approach and MCMC sampling. Migration rate estimates in BayesAss are based on the proportion of individuals in each population that are assigned to other populations with high probability, which can be detected within a few generations. BayesAss allows deviation from Hardy–Weinberg equilibrium but assumes linkage equilibrium, small migration rates and that (sub)population allele frequencies are unaffected by recent genetic drift or migration^64^. We ran BayesAss with 10 million iterations, a sampling frequency of 2,000 and a burn-in of 10%. All other settings were left as default. Ten independent runs with different random seeds were performed to assess MCMC convergence and evaluate the consistency of the results.

### Effects of isolation-by-distance (IBD) and isolation-by-environment (IBE)

We simultaneously tested for potential effects of isolation-by-distance (IBD) and isolation-by-environment (IBE) as an estimate of the impact of urbanization on population differentiation. To this end, we correlated genetic distances [linearized *F*_ST_-values; *F*_ST_/(1 − *F*_ST_)], log-transformed geographic distances, and environmental distances (pairwise differences of the ‘urbanity index’) using multiple matrix regression with randomization analysis (MMRR^65^) in the *ecodist*^66^ package in R using 1,000 permutations. Additionally, we tested for an association between IBD, IBE and migration rate estimates between populations. MMRR has been shown to be robust towards a wide range of dispersal rates and may be preferable to Mantel tests because of more appropriate Type I error rates, and because it ranks variables in terms of their relative effects on genetic distance^65^. To preserve the directional information in the BayesAss migration rates, we ran separate MMRR models on both top and bottom halves of the pairwise matrices (Table 2). In all analyses we used differences in sample sizes as a covariate, under the assumption that sample size reflects population size and thus can affect genetic differentiation.

**Table 2:** Estimates of recent migration rates (*m*) between each population pair, calculated using BayesAss.

	BV	FH	K	OP	RP	IGG	Oskar	BB
BV	**0.6999**	0.0399	0.0417	0.0338	0.0169	0.0292	0.0273	0.0345
FH	0.0232	**0.7113**	0.0392	0.0149	0.0124	0.0136	0.0429	0.0128
K	0.0259	0.0558	**0.7075**	0.0168	0.0236	0.0157	0.0521	0.0163
OP	0.0716	0.0573	0.0699	**0.7987**	0.0153	0.0288	0.0343	0.0489
RP	0.0874	0.0335	0.059	0.0238	**0.8653**	0.1285	0.0383	0.0689
IGG	0.0213	0.0242	0.0183	0.0837	0.0197	**0.7145**	0.077	0.0411
Oskar	0.0196	0.0396	0.0216	0.0145	0.0122	0.0136	**0.6995**	0.0136
BB	0.0512	0.0385	0.0429	0.0138	0.0347	0.0562	0.0286	**0.7638**

Values above the diagonal (top matrix) represent migration rates from the populations in the horizontal row into the populations in the vertical column (e.g., *IGG* into *RP*). Values below the diagonal (bottom matrix) represent migration rates from the populations in the vertical column into the populations in the horizontal row (e.g., *RP* into *IGG*). Values in the diagonal line (highlighted in bold) represent proportions of resident individuals in each population per generation (i.e., non-migrant individuals).

### Effects of urbanization on parameters of genetic diversity and inbreeding

We explored the relationship between the urbanity index and rarefied allelic richness as well as inbreeding coefficients using linear mixed-effects models (LMM). We tested the relationship between the urbanity index and expected and observed heterozygosities by fitting generalized linear mixed-effects models (GLMM) with binomial error structure. This allowed us to control for differences in variability among the examined microsatellite loci by modelling ‘locus ID’ as a random effect. All mixed models were implemented in R using the package *lme4*^67^. We additionally used linear modelling (LM) to test for differences in migration rates between alternative migration routes (rural–urban *vs*. urban–rural and rural–rural *vs*. urban–urban). For this analysis all sites within the city where treated as ‘urban’ and geographic distances between sites served as a covariate. Model assumptions were checked visually. Unless noted otherwise, all statistical analyses were performed in R v. 3.0.2^60^.

### Approval of experimental protocols

All experimental procedures described here were in accordance with the current laws on animal experimentation in Germany and the European Union and approved by licensed hunters (hunting license 1000250221; ID: V54-19c 20/15 152 – F 104/59).

## Results

### Evaluation of microsatellite markers

The number of alleles at each of the ten examined microsatellite loci ranged from 3 to 6, with observed heterozygosities (*H*_o_) per locus ranging from 0.36 to 0.71 and expected heterozygosities (*H*_E_) ranging from 0.55 to 0.76 (Supplementary Table S5). One locus (*sat12*) showed evidence for null alleles and was therefore excluded from subsequent analyses. Significant linkage disequilibrium (LD) was found between *7L1F1* and each of the following loci: *7L1B10*, *7L5A4*, *sat7*, *6L3B4*, and *sat12*. Consequently, we also excluded the *7L1F1* locus from subsequent analyses.

### Genetic diversity, genetic differentiation, bottlenecks and inbreeding

**Figure 2:**
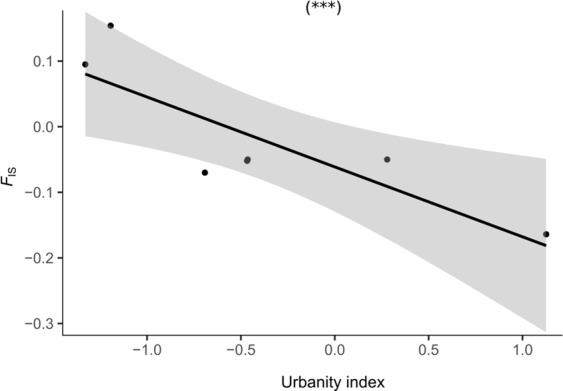
Scatterplot showing the relationship between urbanity indices and inbreeding coefficients (*F*_IS_) in our eight study populations. The lines shows the predicted relationship (linear regression, ***p < 0.001) and the shaded areas the corresponding 95% confidence intervals.

The mean (±SD) allelic richness (*A*_R_), based on rarefaction, was 2.725 ± 0.132 within our entire sample (*N* = 139 individuals) and across loci, while the mean observed heterozygosity (*H*_O_) was 0.553 ± 0.050, and the mean expected heterozygosity (*H*_E_) 0.555 ± 0.038 (Table 1). Global tests of Hardy-Weinberg disequilibrium (i.e., heterozygote deficiencies) were statistically significant only for two of the rural populations (FH and K; *p* < 0.05). We found a significant relationship between the urbanity index and *H*_O_ (GLMM; *χ*^2^ = 4.224, *p* = 0.039) and *F*_IS_ (LMM, *χ*^2^ = 17.008, *p* < 0.001), and *H*_O_ increased, while *F*_IS_ decreased from rural over suburban towards urban populations (Table 1, Fig. 2). The relationships between the urbanity index and *H*_E_ (GLMM, *χ*^2^ = 0.319, *p* = 0.571) and *A*_R_ (LMM, *χ*^2^ = 0.419, *p* = 0.517) were not statistically significant. We did not find evidence for genetic bottlenecks in any of our populations (Wilcoxon signed-rank tests, all *p* > 0.05; Table 1).

Global and pairwise *F*_ST_-values (and the analogous *G*′_ST_-values) supported the idea that populations were significantly differentiated. The global multilocus *F*_ST_-value indicated moderate differentiation across populations (*F*_ST_ = 0.100, 95% CI = 0.085–0.121), as did the global *G*′_ST_ (*G*′_ST_ = 0.208, 95% CI = 0.173–0.243). Most population pairs showed moderate to high genetic differentiation (Supplementary Table S6a,b). Two of the rural populations, K and FH, represent the only population pair that had little genetic differentiation (*F*_ST_ = −0.0067, 95% CI = −0.0417–0.0235; Supplementary Table S6b). The mean (±SD) pairwise *F*_ST_-value among rural populations was 0.031 ± 0.033, 0.119 ± 0.038 among suburban populations, and 0.098 between the two urban study populations.

### Population genetic structure

**Figure 3:**
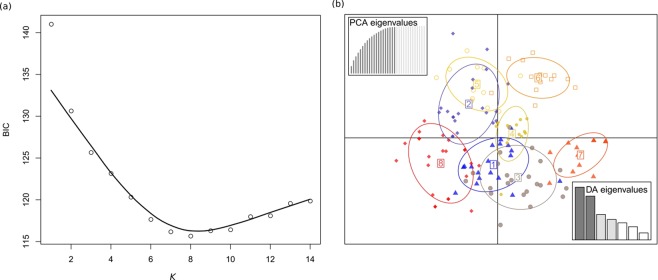
(**a**) Bayesian Inference Criterion (BIC) values versus numbers of clusters (*K*), suggesting that *K* = 8 was the most likely number of genetically distinct clusters. (**b**) Discriminant analysis of principal components (DAPC) scatterplot: 20 PC axes were retained, cumulatively explaining more than 95% of the total variance. Eigenvalues of the analysis are displayed in the inset. Each individual is represented as dots, circles, triangles, or squares, while the suggested clusters are shown as ellipses.

**Figure 4:**
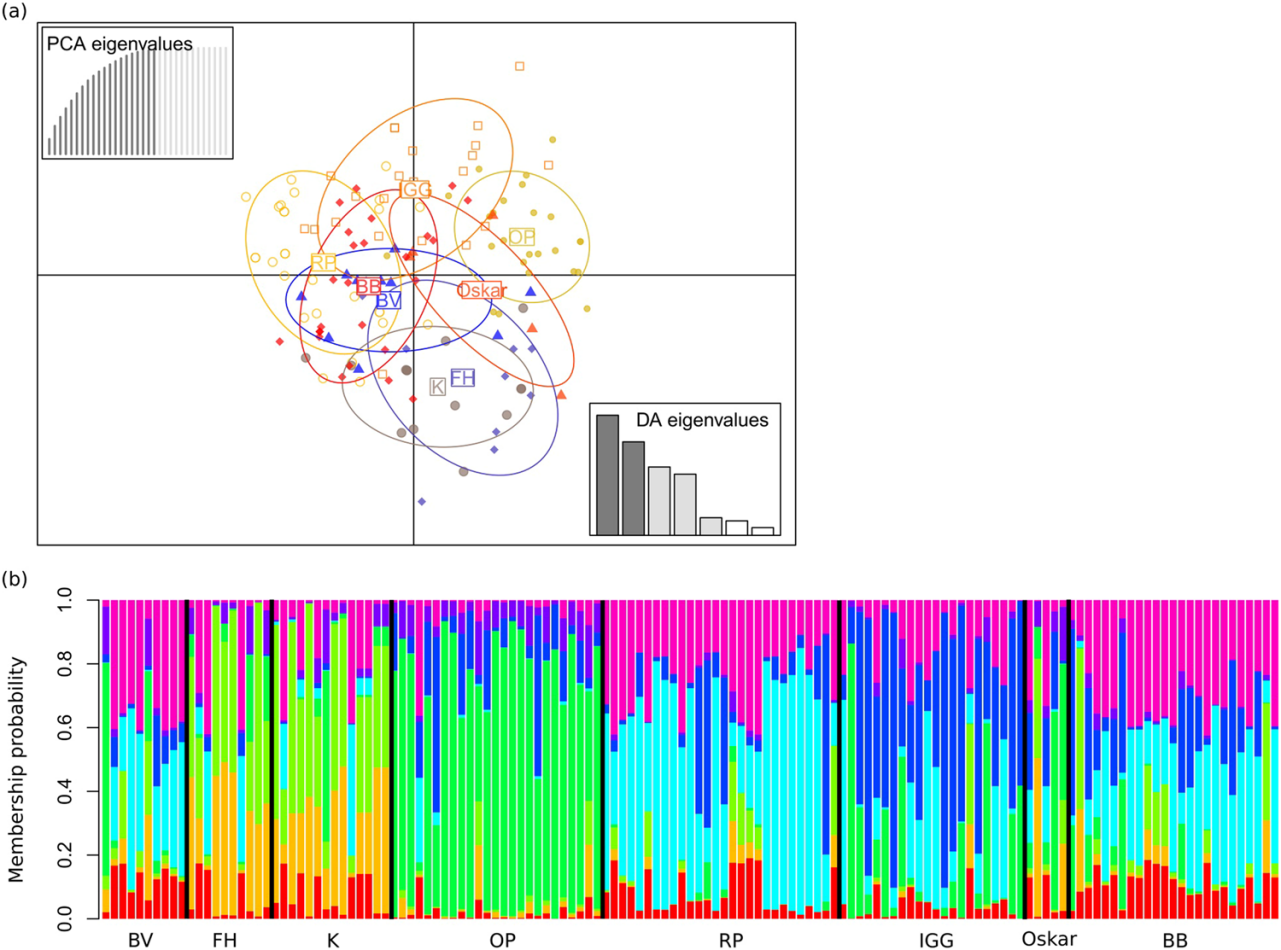
(**a**) Scatterplot showing the genetic distinctiveness among the *N* = 139 individuals based on the first two principal components in our DAPC analysis with prior population assignment. Each individual is represented as dots, circles, triangles, or squares, and the suggested clusters as ellipses. (**b**) Bar plots showing membership probabilities, assessed as the proximity of individuals to different genetic clusters, grouped by population ID.

We first performed DAPC analysis without *a priori* group assignment. To obtain the optimal number of clusters, 20 principal components (PCs) that represented more than 95% of the total variance were retained. The lowest BIC-value (115.70) corresponded to *K* = 8 genetically distinct clusters (Fig. 3). In the second analysis, clusters were defined *a priori* according to the sampling sites. Also in this case, 20 PC axes were retained, cumulatively explaining more than 95% of the total variance, and five discriminant functions were obtained (explaining 98% of the variance; Fig. 3). The DAPC analysis with prior group assignment and subsequent visualization via *compoplot* (i.e., a barplot of membership probability; Fig. 4) uncovered an average assignment probability of 73%. The highest population assignment was estimated for OP (96%), followed by Oskar (80%) and BB (80%). The lowest population assignment was estimated for BV (50%; Supplementary Table S7).

### Migration rates

**Figure 5:**
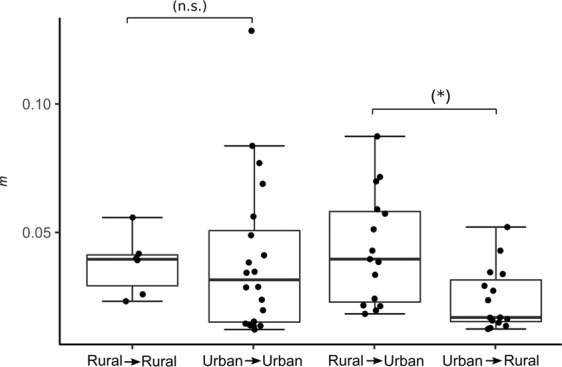
Whisker-box plots of migration rate estimates among rural sites, among urban sites, from rural to urban sites, and from urban to rural sites. *n.s*., not significant; *p < 0.05.

Estimates of contemporary migration obtained from BayesAss suggest high self-recruitment rates (i.e., high proportions of resident, non-migrant individuals) for all populations (Table 2). The highest migration rate occurred out of the urban IGG into the suburban RP site (migration rate; *m* = 0.129; Table 2). Migration rates did not differ when comparing the potential migration directions ‘rural–rural’ and ‘urban–urban’ (LM, *t* = −0.599, *p* = 0.797; Fig. 5). Nonetheless, migration rates were significantly higher for the route ‘rural–urban’ than for ‘urban–rural’ (LM, *t* = 2.763, *p* = 0.015; Fig. 5).

### Isolation-by-distance and isolation-by-environment

**Figure 6:**
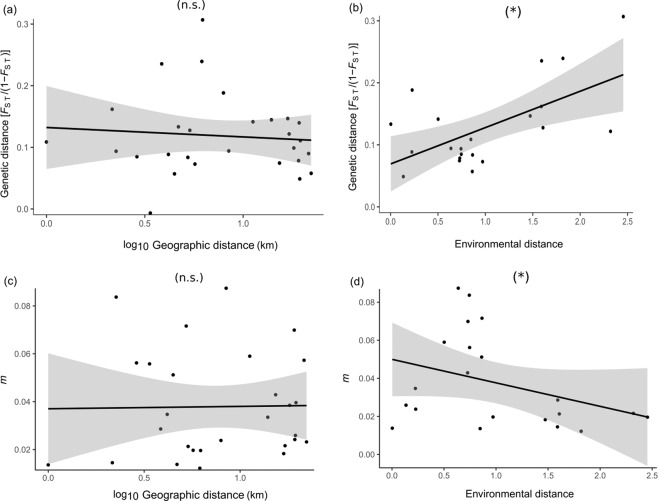
Relationship between pairwise genetic distances, expressed as *F*_ST_/(1 − *F*_ST_) and (**a**) log-transformed geographic distance and (**b**) environmental distances (differences in urbanity). We further show the relationships between recent migration rates (estimated using BayesAss) and (**c**) log-transformed geographic distances and (**d**) environmental distances. Lines show predicted relationships (linear regressions) and shaded areas the corresponding 95% confidence intervals. *n.s*., non-significant; *p < 0.05.

We did not find any evidence for isolation-by-distance (IBD), as the effect of geographic distance on genetic differentiation was not statistically significant (MMRR, ϐ = 0.003, *p* = 0.868; Fig. 6a). Also, sample size (a rough proxy for population size) was a poor predictor of genetic distances (ϐ = −0.008, *p* = 0.542). We did, however, find evidence for isolation-by-environment (IBE), as pairwise environmental distances (based on the urbanity index) was a significant predictor of genetic distance (ϐ = 0.049, *p* = 0.018; Fig. 6b).

Isolation by distance (IBD) did not explain migration rates estimated from BayesAss (MMRR, above diagonal, ϐ = −0.004, *p* = 0.603; below diagonal: ϐ = −0.006, *p* = 0.327; Fig. 6c). By contrast, the IBE model revealed a significant effect in the bottom matrix of BayesAss migration estimates (above diagonal, ϐ = 0.001, *p* = 0.771; below diagonal, ϐ = −0.014, *p* = 0.016; Fig. 6d).

## Discussion

Despite the rapid decline of European rabbit populations across Europe^45^, population genetic studies assessing potential migration routes and gene flow between declining rural populations and populations that (so far) were thriving in urban areas have not yet been conducted. Here, by assessing allele length polymorphisms of ten nuclear microsatellite markers and employing a set of population genetic analyses, we investigate the genetic diversity, population genetic structure, and recent migration among *O. cuniculus* populations across a rural-to-urban gradient in Frankfurt am Main in Germany. Observed heterozygosity (*H*_O_) increased, while the inbreeding coefficient (*F*_IS_) decreased with increasing levels of urbanization (i.e., towards the city centre). Our microsatellite data also revealed significant genetic structure among the studied populations, and models assuming isolation-by-environment (IBE)—based on urbanization effects—explained a greater proportion of both pairwise genetic differentiation (*F*_ST_) and migration rates (*m*) than models assuming isolation-by-distance (IBD). We argue that rabbit populations in rural and urban areas derived from the same ancestral populations that were present as early as 1827^50^ and successfully colonized the inner city in 1930 (Stadtarchiv Frankfurt am Main). This migration pattern is still observable today, since we found higher migration rates from rural into urban populations than vice versa.

Several studies documented a loss of heterozygosity and reduced allelic richness at microsatellite loci in animal and plant populations inhabiting urban areas^4^. By contrast, urbanization in the metropolitan area of Frankfurt and the associated typical changes in landscape structure and habitat types (e.g., mosaic-like land-use patterns) seem to have a neutral or even a positive effect on the genetic diversity of European rabbit populations, as our analyses revealed higher heterozygosity and reduced inbreeding with increasing urbanization. However, our results are not surprising for an ‘urban adaptor’—such as the European rabbit—that during our sampling period occurred at high population densities in German cities^17,26,46,49^. Habitat heterogeneity, increased availability of resources, and low densities (or absence) of predators and competitors are among the hypothesized factors explaining the successful colonisation of cities by European rabbits^49^. Our results are in line with a previous study on a small mammalian ‘urban adaptor’, the white-footed mouse (*Peromyscus leucopus*), in New York City^68^. Munshi-South & Kharchenko^68^ found high genetic diversity, measured as heterozygosity, in urban populations, most likely explained by the high population densities in urban forest fragments.

The distribution and abundance of rural rabbit populations are determined to a considerable extent by habitat heterogeneity, such as the proportion and type of canopy cover, availability of shrubs, and the general plant species composition^69^. Previous studies reported high population densities in scrublands and areas characterized by interspersed patches of natural vegetation and crops^39,70^. Such landscapes provide a combination of both food and refuge for rabbits^39^. However, rural landscapes in Europe are becoming increasingly less structurally-complex and functionally-diverse due to a general homogenisation of agro-ecosystems and fragmentation of remaining patches of less intensely used areas^71^. This trend leads to the widespread loss of important habitats and the necessary landscape configuration required by European rabbits^39,72^ and is likely to be a major cause of the on-going decline of rabbit populations in rural parts of Europe^39^. The rural sites sampled for our present study are prime examples of homogenized and structurally impoverished open landscapes. In a previous study that included the same study sites, Ziege *et al*.^46^ found considerably lower population densities in our rural (0.80 individuals ha^−1^) compared to the urban (14.72 individuals ha^−1^) and suburban study areas (8.51 individuals ha^−1^). The small remnant populations in rural areas are likely prone to loss of genetic diversity due to drift and inbreeding.

We found substantial genetic structure among populations, as our analyses of population genetic differentiation (*F*-statistics) among all sampled populations revealed deviation from panmixia, and all urban populations were genetically differentiated from each other. This was also supported by the *K-means* clustering and discriminant analysis of principal components (DAPC), in which eight genetically distinct clusters were identified with relatively high assignment probabilities for our predefined populations ($\bar{x}$ ± SD = 0.70 ± 0.14) and by the *BayesAss* migration estimates, which showed high proportions of non-migrant individuals in each population and generation ($\bar{m}$ ± SD = 0.74 ± 0.05), rendering human-mediated translocations between sampling localities an unlikely scenario. This pattern seems to be at odds with high estimates of genetic diversity in urban populations (see above). We argue that not enough time has elapsed to bring about a reduction of allelic richness and heterozygosity in the rather isolated urban populations. The combination of substantial genetic differentiation but high genetic diversity was also documented in the white-footed mouse^68^ and may be a general characteristic of ‘urban adapters’ with poor dispersal capabilities.

European rabbits change their dispersal behaviour depending on local ecological conditions. Dispersal increases at low population densities, while philopatry prevails when population densities are high, whereby males show more dispersal than females^73^. Estimated dispersal distances are relatively small (with median distances of 460 m for males and 232 m for females^73,74^). However, studies on Australian *O. cuniculus* populations demonstrated the capability of this species to move more than 20 km^75^. A recent study on home ranges of seven females and six males in two of the study sites included here, the suburban site RP (*Rebstockpark*) and the urban site *Friedberger Anlage* (belonging to IGG), revealed exceedingly small home ranges, with a ranging radius of less than 50 m, at both sites (Ziege *et al*. submitted). The results of our present study, therefore, support the general notion of limited dispersal behaviour in rabbits^73,74^. Relatively higher dispersal at low population densities could explain the comparatively lower genetic differentiation and higher migration rates among our rural study populations, as well as the asymmetric (albeit low) migration rates from rural into urban populations.

Multiple matrix regression with randomization (MMRR) revealed that isolation-by-environment (IBE)—based on differences in the degree of urbanization differences among our study sites—but not isolation-by-distance (IBD) explained the observed population genetic differentiation and variation in migration rates among populations.

Overall, our results suggest that urbanization has played an important role in shaping the spatial distribution of genetic variation of European rabbit populations in and around Frankfurt am Main. Our results are in line with meta-analyses^35,76^ that uncovered a high prevalence of IBE in natural systems. The patterns of IBE observed here can be explained by the action of natural selection in urban environments and/or non-random mating or migration associated with urbanization. The absence of IBD indicates that genetic patterns across our study populations are unlikely to be the product of drift and dispersal limitation alone. Reduced representation genome sequencing (e.g., RAD-sequencing^77^) across several independent, replicated urbanization gradients spanning the distribution range of European rabbits will be necessary to robustly assess genome-wide genetic diversity at thousands of SNPs and to partition genetic diversity into neutral and adaptive diversity. Such an approach will also allow identifying both key demographic parameters (using neutral markers) and candidate loci under selection in urban populations.

The demographic changes and transformation of land-use characterising the Anthropocene will likely continue for the next decades^1,78^. This highlights the need for empirical data on the influence of cities on the ecology and evolution of species that live side-by-side with humans, which could provide the basis for future conservation and management plans. The results presented here are in line with previous studies suggesting that urban habitats can support high populations of European rabbits^46,49^. Nonetheless, we also identified urbanization as a strong driver of genetic differentiation. The substantial genetic structure observed in urban populations, in combination with high genetic diversity, likely reflects high population densities but limited dispersal abilities in urbanized areas, or simply reduced dispersal behaviour. Habitat corridors that promote the connectivity of green areas both within cities and to the rural outskirts might enhance gene flow and help maintain the genetic diversity of urban populations, which may become vital in the future conservation of this species.

### Limitations of our study and alternative interpretations of our results

Our findings are largely consistent with previous studies on population monitoring of European rabbits in the region^46,49^. A major limitation of our present study, however, results from the small number of neutral microsatellite markers employed in our study. Another potential caveat is that the population genetic patterns observed here could be due to released or escaped domesticated rabbits interbreeding with wild European rabbits. However, we argue that this is highly unlikely to happen for two main reasons: first, survival and reproduction rates of released domestic rabbits are considerably lower than those of wild rabbits, as domestic rabbits are more susceptible to predation^79^. Second, during our field work (2010–2015), we observed only one individual with a fur colouration characteristic of domestic rabbits amongst hundreds of wild rabbits that were monitored^46,48,49^. This individual was easy to distinguish from wild rabbits as it was considerably smaller, with an entirely black fur. If different levels of interbreeding between pet and wild rabbits would be the main driver behind the patterns described in our present study, this should visibly affect phenotypes; e.g. hybrid individuals should differ in size (smaller than wild rabbits), behaviour (tamer than wild rabbits), and especially fur coloration (with black coloration being dominant in crossbreeds)^80,81^. Based on these criteria, none of the studied populations showed any indication of interbreeding.

Finally, we cannot exclude the possibility that rabbits were (illegally) relocated within our study region. Although relocation of European rabbits in the federal state of Hessia is forbidden by law, some hunters might still have moved individuals between populations so as to enhance their hunting grounds. Relocations could have occurred between urban donor and rural recipient populations. Nonetheless, we argue that potential relocations had a minor influence on the observed population genetic patterns due to the rarity of such events. Moreover, translocation success is low owing to the strong territorial behaviour and matrilineal social organization of European rabbits^82^. Finally, we found significant genetic differentiation between all studied populations, making it unlikely that repeated translocations between them occurred.

## Supplementary information

Table S1; Table S2; Table S3; Table S4; Table S5; Table S6a,b; Table S7.

## Data Availability

Microsatellite data (Genepop file format) are available as supplementary information material.
