# Supplementary material for: Population genetics of the European rabbit along a rural-to-urban gradient
Source: Sci Rep. 2020 Feb 12;10:2448. doi: 10.1038/s41598-020-57962-3 (PMC7015939; doi:10.1038/s41598-020-57962-3)
Supplement: Supplementary file 1 — Table S1; Table S2; Table S3; Table S4; Table S5; Table S6a,b; Table S7. [file 41598_2020_57962_MOESM1_ESM.docx]

**Supplementary Material**

**Population genetics of the European rabbit along a rural-to-urban gradient**

Madlen Ziege^1*^, Panagiotis Theodorou^2^, Hannah Jüngling^3^, Stefan Merker^4^, Martin Plath^5^, Bruno Streit^6^, and Hannes Lerp^7^

^1^ University of Potsdam, Plant Ecology and Nature Conservation, Am Mühlenberg 3, D-14476 Potsdam (Golm), Germany; madlen.ziege@uni-potsdam.de

^2^ Martin-Luther-University Halle-Wittenberg, Institute of Biology, General Zoology, Hoher Weg 8, D-06120 Halle (Saale), Germany; panatheod@gmail.com

^3^ Senckenberg Gesellschaft für Naturforschung, Clamecystraße 12, D-63571 Gelnhausen, Germany; juengling.han@gmail.com

^4^ State Museum of Natural History Stuttgart, Department of Zoology, Rosenstein 1, D-70191 Stuttgart, Germany; stefan.merker@smns-bw.de

^5^ Northwest A&F University, College of Animal Science and Technology, Yangling, Shaanxi 712100, China; [mplath-zoology@gmx.de](mailto:martin_plath@web.de)

^6^ University of Frankfurt, Department of Ecology & Evolution, Max-von-Laue-Str. 13, D-60438, Frankfurt am Main, Germany; streit@bio.uni-frankfurt.de

^7^ Museum Wiesbaden, Natural History Collections, Friedrich-Ebert-Allee 2, D-6518 Wiesbaden, Germany; hannes.lerp@gmx.de

* Corresponding author:

Madlen Ziege, Phone: +49 331 977 6254; Fax: +49 331 977 1930; Email: madlen.ziege@mailbox.org

**Table S1.** Pairwise geographic distances (km) between the *Oryctolagus cuniculus* populations.

|  | BV | FH | K | OP | RP | IGG | Oskar | BB |
| --- | --- | --- | --- | --- | --- | --- | --- | --- |
| BV | 0 |  |  |  |  |  |  |  |
| FH | 22.28 | 0 |  |  |  |  |  |  |
| K | 19.43 | 3.36 | 0 |  |  |  |  |  |
| OP | 5.21 | 21.50 | 19.19 | 0 |  |  |  |  |
| RP | 8.49 | 13.95 | 11.37 | 7.93 | 0 |  |  |  |
| IGG | 5.37 | 19.27 | 16.92 | 2.26 | 5.67 | 0 |  |  |
| Oskar | 6.21 | 19.42 | 17.21 | 2.16 | 6.15 | 0.99 | 0 |  |
| BB | 4.46 | 18.07 | 15.39 | 4.69 | 4.12 | 2.90 | 3.85 | 0 |

**Table S2.** Microsatellite loci used in our study.

| Microsatellite locus | Repeat motif | Allelic size range (bp) | Reference |
| --- | --- | --- | --- |
| 5L1C3 | [CA]12 | 188-194 | 2 |
| sat13 | [GT]13 | 114-128 | 1 |
| 7L1B10 | [CA]12 | 193-199 | 2 |
| sat16 | [TG]15 | 109-115 | 1 |
| 7L5A4 | [CA]13 | 146-150 | 2 |
| 12L1E11 | [CA]13 | 231-238 | 2 |
| 7L1F1 | [AG]9[CA]11 | 141-160 | 2 |
| sat7 | [TG]14 | 184-195 | 1 |
| 6L3B4 | [CT]9[CA]17 | 134-157 | 2 |
| sat12 | [CTAT]10 | 122-138 | **1** |

^1^(Mougel *et al.* 1997), ^2^(Korstanje *et al.* 2003)

**Table S3.** List of microsatellite loci used in our study and their annealing temperatures (T_m_).

| Microsatellite locus | Primer 5′-3′ | T_m_  (°C) |
| --- | --- | --- |
| 5L1C3 | F:CAGCGGTAAGAGTGAGAAAC | 60 |
|  | R:TCCCCCATAACAAAAGAGG |  |
| sat13 | F:CAGTTTTGAAGGACACCTGC | 55 |
|  | R:GCCTCTACCTTTGTGGGG |  |
| 7L1B10 | F:TTGGCAGGAAGAAAAGGAAGATT | 60 |
|  | R:TTTGTCATAAGCATTTGGGAAGTG |  |
| sat16 | F:AATCAGCCTCTATGAATTCCC | 55 |
|  | R:AATGCTACATGGTAACCAGGC |  |
| 7L5A4 | F:TAGTGAGCCTCTAACTTCTGTA | 60 |
|  | R:CCCTAACATGGGGAAATG |  |
| 12L1E11 | F:AGTGGTAGCGCTTTGGTCTG | 60 |
|  | R:GCTCCTTGGGGCATTTG |  |
| 7L1F1 | F:ACACCTGGGGAATAAACAACAAG | 60 |
|  | R:GAGGGAGGCAGAGGGATAAGA |  |
| sat7 | F:GTAACACCCATGCACACTC | 60 |
|  | R:GCACAATACCTGGGATGTAG |  |
| 6L3B4 | F:CGAGCTCCTTGCTGCATGAC | 60 |
|  | R:AGGGCGACCAGCGGTCTAT |  |
| sat12 | F:CTTGAGTTTTAAATTCGGGC | 55 |
|  | R:GTTTGGATGCTATCTCAGTCC |  |

**Table S4.** Configuration of the multiplex mix.

| Oligonucleotide mix 1 | | | |
| --- | --- | --- | --- |
| Microsatellite locus | Size (bp) | Fluorescent tag | |
|  |  | Dye colour | 5′-labeling |
| 5L1C3 | 188-194 | Black | Biomers DY-751 |
| sat13 | 114-128 | Black | Biomers DY-751 |
| 7L1B10 | 193-199 | Blue | Metabion CY5-K |
| sat16 | 109-115 | Green | Metabion IRD700 |
| 7L5A4 | 146-150 | Green | Metabion IRD700 |
| Oligonucleotide mix 2 | | | |
| Microsatellite locus | Size (bp) | Fluorescent tag | |
|  |  | Dye colour | 5′-labeling |
| 12L1E11 | 231-238 | Black | Biomers DY-751 |
| 7L1F1 | 141-160 | Black | Biomers DY-751 |
| sat7 | 184-195 | Blue | Metabion CY5-K |
| 6L3B4 | 134-157 | Blue | Metabion CY5-K |
| sat12 | 122-138 | Green | Metabion IRD700 |

**Table S5.** Number of alleles (*N*_A_), observed (*H*_OBS_) and expected (*H_EXP_*) heterozygosity, and Wright’s fixation index (*F*_IS_) of 10 microsatellite loci within populations of the *Oryctolagus cuniculus*. Values in bold indicate significant departure from HWE, after Bonferroni correction. Locus sat12 was not included in further analyses, as it showed evidence of null alleles.

| Locus | *N*_A_ | *H*_OBS_ | *H*_EXP_ | *F*_IS_ |
| --- | --- | --- | --- | --- |
| 5L1C3 | 6 | 0.49 | 0.56 | 0.10 |
| sat13 | 3 | 0.42 | 0.55 | 0.08 |
| 7L1B10 | 6 | 0.64 | 0.66 | -0.02 |
| sat16 | 6 | 0.48 | 0.69 | 0.26 |
| 7L5A4 | 5 | 0.53 | 0.61 | -0.02 |
| 12L1E11 | 4 | 0.64 | 0.62 | -0.06 |
| 7L1F1 | 4 | 0.56 | 0.69 | 0.13 |
| sat7 | 6 | 0.62 | 0.67 | -0.04 |
| 6L3B4 | 5 | 0.71 | 0.67 | -0.09 |
| sat12 | 5 | 0.36 | 0.76 | **0.51** |
| All loci (mean) | 5 | 0.54 | 0.64 | 0.08 |

**Table S6a.** Average pairwise *F*_ST_ for *Oryctolagus cuniculus* among the eight sampling sites estimated using the *diversity* R package

|  | BV | FH | K | OP | RP | IGG | Oskar | BB |
| --- | --- | --- | --- | --- | --- | --- | --- | --- |
| BV | 0 |  |  |  |  |  |  |  |
| FH | 0.054687 | 0 |  |  |  |  |  |  |
| K | 0.046533 | -0.00674 | 0 |  |  |  |  |  |
| OP | 0.077265 | 0.082485 | 0.072747 | 0 |  |  |  |  |
| RP | 0.086151 | 0.126332 | 0.123946 | 0.158506 | 0 |  |  |  |
| IGG | 0.113176 | 0.122609 | 0.127917 | 0.085679 | 0.0679 | 0 |  |  |
| Oskar | 0.234802 | 0.099815 | 0.108519 | 0.139269 | 0.193169 | 0.098073 | 0 |  |
| BB | 0.053934 | 0.090042 | 0.069325 | 0.117656 | 0.081324 | 0.07825 | 0.190585 | 0 |

**Table S6b.** Pairwise *F*_ST_ for *Oryctolagus cuniculus* among the eight sampling sites including lower and upper 95% CI.

| comparison | BC_mean | BC_Lower_95%CI | BC_Upper_95%CI |
| --- | --- | --- | --- |
| BV, vs. FH, | 0.0547 | 0.0036 | 0.1189 |
| BV, vs. K, | 0.0465 | 0.0066 | 0.0857 |
| BV, vs. OP, | 0.0773 | 0.0286 | 0.1166 |
| BV, vs. RP, | 0.0862 | 0.0424 | 0.1292 |
| BV, vs. IGG, | 0.1132 | 0.0816 | 0.1637 |
| BV, vs. Oskar, | 0.2348 | 0.1801 | 0.2802 |
| BV, vs. BB, | 0.0539 | 0.017 | 0.0908 |
| FH, vs. K, | -0.0067 | -0.0417 | 0.0235 |
| FH, vs. OP, | 0.0825 | 0.0557 | 0.1063 |
| FH, vs. RP, | 0.1263 | 0.0839 | 0.1779 |
| FH, vs. IGG, | 0.1226 | 0.0852 | 0.187 |
| FH, vs. Oskar, | 0.0998 | 0.0122 | 0.1641 |
| FH, vs. BB, | 0.09 | 0.0541 | 0.1343 |
| K, vs. OP, | 0.0727 | 0.052 | 0.0958 |
| K, vs. RP, | 0.1239 | 0.0811 | 0.1817 |
| K, vs. IGG, | 0.1279 | 0.1005 | 0.1528 |
| K, vs. Oskar, | 0.1085 | 0.0418 | 0.1713 |
| K, vs. BB, | 0.0693 | 0.039 | 0.1043 |
| OP, vs. RP, | 0.1585 | 0.1452 | 0.1877 |
| OP, vs. IGG, | 0.0857 | 0.0699 | 0.1092 |
| OP, vs. Oskar, | 0.1393 | 0.1154 | 0.1923 |
| OP, vs. BB, | 0.1177 | 0.1017 | 0.1386 |
| RP, vs. IGG, | 0.0679 | 0.0435 | 0.0964 |
| RP, vs. Oskar, | 0.1932 | 0.1558 | 0.2189 |
| RP, vs. BB, | 0.0813 | 0.0666 | 0.1009 |
| IGG, vs. Oskar, | 0.0981 | 0.0437 | 0.167 |
| IGG, vs. BB, | 0.0782 | 0.0498 | 0.1161 |
| Oskar, vs. BB, | 0.1906 | 0.1449 | 0.242 |
|  |  |  |  |

**Table S7**. Population assignment based on the DAPC analysis.

| Site | Urbanity | Population assignment (%) |
| --- | --- | --- |
| BV | -1.329 | 0.5 |
| FH |  | 0.6 |
| K | -1.194 | 0.652 |
| OP | -0.464 | 0.96 |
| RP | -0.692 | 0.75 |
| IGG | 0.279 | 0.59 |
| Oskar | 1.1126 | 0.8 |
| BB | -0.467 | 0.8 |

**References**

Korstanje R, Gillissen GF, Versteeg SA *et al.* (2003) Mapping of Rabbit Microsatellite Markers Using Chromosome-Specific Libraries. *Journal of Heredity*, **94**, 161–169.

Mougel F, Mounolou JC, Monnerot M (1997) Nine polymorphic microsatellite loci in the rabbit, Oryctolagus cuniculus. *Animal genetics*, **28**, 58–9.
